# Mechanistic and structural basis for inhibition of thymidylate synthase ThyX

**DOI:** 10.1098/rsob.120120

**Published:** 2012-10

**Authors:** Tamara Basta, Yap Boum, Julien Briffotaux, Hubert F. Becker, Isabelle Lamarre-Jouenne, Jean-Christophe Lambry, Stephane Skouloubris, Ursula Liebl, Marc Graille, Herman van Tilbeurgh, Hannu Myllykallio

**Affiliations:** 1 Laboratoire d'Optique et Biosciences, INSERM U696, CNRS UMR 7645, Ecole Polytechnique, Palaiseau Cedex, Palaiseau 91228, France; 2 Mbarara Research Base, Epicentre, PO Box 1956 Mbarara, Uganda; 3 UPMCUniv Paris 06, 75005 Paris, France; 4 Institut de Biochimie et de Biophysique Moléculaire et Cellulaire, CNRS UMR 8619, Université Paris-Sud, Orsay 91405, France; 5 Université Paris-Sud, Orsay 91405, France

**Keywords:** thymidylate synthase ThyX, inhibitor screen, antimicrobial compounds, naphthoquinone

## Abstract

Nature has established two mechanistically and structurally unrelated families of thymidylate synthases that produce de novo thymidylate or dTMP, an essential DNA precursor. Representatives of the alternative flavin-dependent thymidylate synthase family, ThyX, are found in a large number of microbial genomes, but are absent in humans. We have exploited the nucleotide binding pocket of ThyX proteins to identify non-substrate-based tight-binding ThyX inhibitors that inhibited growth of genetically modified *Escherichia coli* cells dependent on *thyX* in a manner mimicking a genetic knockout of thymidylate synthase. We also solved the crystal structure of a viral ThyX bound to 2-hydroxy-3-(4-methoxybenzyl)-1,4-naphthoquinone at a resolution of 2.6 Å. This inhibitor was found to bind within the conserved active site of the tetrameric ThyX enzyme, at the interface of two monomers, partially overlapping with the dUMP binding pocket. Our studies provide new chemical tools for investigating the ThyX reaction mechanism and establish a novel mechanistic and structural basis for inhibition of thymidylate synthesis. As essential ThyX proteins are found e.g. in *Mycobacterium tuberculosis* and *Helicobacter pylori*, our studies have also potential to pave the way towards the development of new anti-microbial compounds.

## 2. Introduction

**Figure 1.**
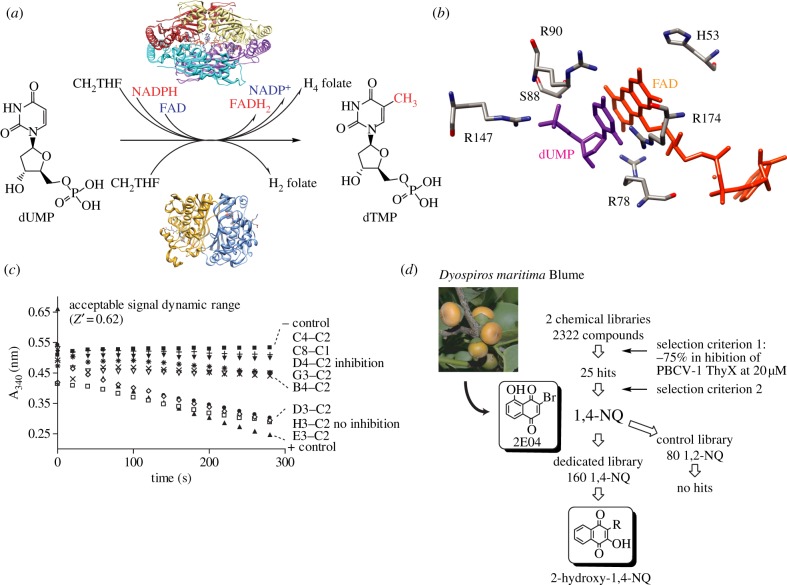
Screening process for identification of ThyX inhibitors. (*a*) The last step in the de novo synthesis of thymidylate is catalysed by two unrelated families of thymidylate synthases, ThyX (top) and ThyA (bottom). (*b*) Unique stacking interaction of the pyrimidine ring of dUMP (magenta) with the isoalloxazine ring of the FAD cofactor at the active site pocket of *T. maritima* ThyX. Conserved active-site residues of ThyX proteins are shown. (*c*) Validation of the ThyX activity assay based upon detection of the NADPH oxidation, followed by decrease of A_340_. Negative control without enzyme and positive control with PBCV-1 ThyX are shown. Also shown are representative data indicating inhibition of ThyX activity or lack thereof. Inhibition parameters for specific molecules are given in table 1 and the main text. (*d*) Flow chart describing distinct steps leading to the identification of 2-hydroxy-1,4-naphthoquinones as specific ThyX inhibitors. Selection criteria 1 and 2 are described in the text. The lead compound 2-bromo-5-hydroxy-1,4-naphthoquinone (2E04) was isolated from the fruit of the Malaysian persimmon (*Dyospiros maritima* Blume); NQ, naphthoquinone.

De novo 2′-deoxythymidine-5′-monophosphate (dTMP or thymidylate) synthesis is a well-established target for inhibiting cellular growth [1–3]. The last step of dTMP synthesis is the methylation of 2′-deoxyuridine-5′-monophosphate (dUMP) to dTMP. This N^5^,N^10^-methylene-5,6,7,8-tetrahydrofolate (CH_2_H_4_folate)-dependent reaction is catalysed by two distinct families of thymidylate synthases, ThyA (EC 2.1.1.45) and ThyX (EC 2.1.1.148; flavin-dependent thymidylate synthase (FDTS)), without detectable sequence [4] or structural [5–8] similarity (figure 1*a*). Whereas human and bacterial ThyA proteins use tetrahydrofolate (H_4_folate) to reduce the methylene moiety after its transfer to the uracil ring, ThyX proteins use a non-covalently bound flavin adenine dinucleotide (FAD) cofactor to facilitate hydride transfer from NAD(P)H [9–11]. Recent studies have demonstrated accumulation of a 5-hydroxymethyl-dUMP (5hmdUMP) as an acid-trapped intermediate during ThyX catalysis [10]. Interestingly, 5hmdUMP stoichiometrically replaces dTMP in the genome of the bacteriophage SPO1 of *Bacillus subtilis* [12]. Strikingly, this modified nucleotide is synthetized by a viral protein homologous to thymidylate synthase ThyA [13,14].

Although details of the ThyX reaction mechanism are not fully established, the pronounced structural and mechanistic differences between ThyA and ThyX proteins provide an excellent starting point for investigating how two distinct ways of producing thymidylate have evolved and how the activities of these enzymes may be regulated and controlled in a cellular setting. The fact that the ThyX homotetramer does not show significant structural similarity to any other protein structure currently known reveals the uniqueness of ThyX proteins. On the other hand, the different ThyX proteins share a high level of structural similarity, as exemplified by a *Z*-value of 18.0 between the *Paramecium bursaria chlorella* virus (PBCV-1) and *Mycobacterium tuberculosis* ThyX proteins. Site-directed mutagenesis studies together with several ThyX crystal structures revealed that the active site of tetrameric ThyX proteins (figure 1*b*) is located at the interface of three monomers, allowing surface exposure of the N5 atom of the isoalloxazine ring participating in hydride transfer [5,6,8,15]. The large active site cavity is formed at the centre of the tetramer that contains the FAD cofactor. ThyX proteins do not carry a specific domain, such as a Rossman fold, for fixation of nicotine adenine dinucleotide phosphate, reduced form (NADPH). As particular microaerophilic conditions are necessary for observing genetic complementation of an *E. coli* *ΔthyA* strain by *Campylobacter jejuni thyX* [16], this hydride transfer is likely inhibited by the presence of molecular oxygen.

ThyX has a complex fold with a central a/b domain flanked by two helical domains. It forms a tetramer with a 222 symmetry. For most of the documented structures, ThyX has FAD bound in an extended conformation and with the adenine ring buried in a deep binding pocket in the enzyme. The key feature of the active site of ThyX proteins is the stacking of the pyrimidine ring of dUMP against the isoalloxazine ring of the FAD cofactor [5,6,8]. This interaction is of particular interest as dUMP functions as activator of the NAD(P)H oxidase activity of PBCV-1 (activating factor >20 [17]) and *Thermotoga maritima* (activating factor ≈ 5–7) ThyX proteins. Previous steady-state kinetic analyses have suggested the formation of a ternary NADPH–dUMP–ThyX complex during catalysis [5,17]. Early kinetic studies indicated that CH_2_H_4_folate competitively inhibits NADPH oxidase activity of the PBCV-1 ThyX protein [5,17], suggesting that folate and NADPH binding sites of ThyX proteins overlap. Indeed, this notion is supported by the fact that docking of the NADPH to the crystallographically defined folate binding site of ThyX proteins is feasible [18]. A flexible loop in the vicinity of the active site contributes to the binding of dUMP and is likely to undergo conformational changes during catalysis [6,8]. The key residues that form a direct hydrogen bond with dUMP (for instance Arg-78, Arg-80, Ser-88 and Arg-90 in *T. maritima* ThyX) are conserved in the ThyX protein family, but not in other dUMP binding proteins [5,6,8,19]. Thus, the binding characteristics of dUMP and its role as catalytic activator define the nucleotide binding site as a unique feature of ThyX proteins. Some dUMP analogues have been identified as ThyX inhibitors that may bind weakly to the nucleotide binding pocket of ThyX proteins [20–22].

Several arguments underline that, in addition to answering mechanistic questions, identification and development of specific ThyX inhibitors is needed. Considering their crucial metabolic role in bacterial cells, ThyX proteins have been proposed as a priority target for developing new anti-microbial compounds [3,4]. Notably, *thyX* can be deleted only in the presence of thymidine kinase Tdk, an enzyme that salvages extracellular thymidine, thus providing in few cases a metabolic by-pass for thymidylate synthase [23]. Many important bacterial pathogens carry *thyX*, but lack *tdk* (for a listing, see the electronic supplementary material, table S1). *Mycobacterium* strains are peculiar due to the presence of both *thyX* and *thyA* genes, but even in this case, *thyX* has been shown to code for essential cellular function(s) [24]. Moreover, in other Corynebacteriaceae, ThyX proteins have been specifically implicated in survival during the stationary growth phase [25].

In order to identify selective ThyX inhibitors, we have performed an efficient activity-based screen that identified a considerable number of non-substrate based ThyX inhibitors that do not act on human thymidylate synthase. Our data established that preventing the binding of dUMP to the ThyX active site markedly inhibited NAD(P)H-oxidase activity of ThyX proteins. The co-crystal structure of a 2-hydroxy-1,4-naphthoquinone (2-OH-1,4-NQ) compound with PBCV-1 ThyX indicated an unexpected binding configuration within the conserved active site of the tetrameric ThyX enzyme, at the interface of two ThyX monomers that is distinct from earlier described structural data of ThyX-substrate/inhibitor complexes [6,8]. Moreover, our study provides a proof of concept for the selective inhibition of ThyX proteins and encourages further development of tight-binding ThyX inhibitors towards possible biomedical uses.

## 3. Material and methods

### 3.1. Chemicals

All chemicals were from Sigma unless otherwise indicated.

### 3.2. Bacterial strains and plasmids

*E. coli* BL21 (F-, *ompT*, *hsdS* (r_B_-,m_B_-) *gal* *dcm*) (Novagen) was used for protein expression in Luria Bertani (LB) medium containing 100 µg ml^−1^ ampicillin. *Helicobacter pylori* ThyX expression was induced using 0.2 per cent arabinose [4]. Expression of all other proteins was achieved using 1 mM isopropyl β-d-thiogalactopyranoside (IPTG). *Mycobacterium tuberculosis* ThyX was expressed and purified using previously published protocols [19]. Human thymidylate synthase expression used a codon-optimized synthetic gene construct previously constructed (GenBank accession no. EU520475.1). *Chlamydia trachomatis thyX* was PCR amplified using chromosomal DNA and cloned into pQEL-80 expression plasmids during this work.

### 3.3. Medium throughput inhibitor screen

The NADPH oxidation assay for PBCV-1, *C. trachomatis* and *M. tuberculosis* ThyX activity [16] was adapted for a medium throughput inhibitor screen (MTS) in 96-well plates [21]. 1982 compounds from the National Cancer Institute diversity set I and 340 natural compounds, selected for their diversity and drug-likeness (GreenPharma), were added to a final concentration of 20 µM in the initial screen. All molecules were solubilized using dimethylsulfoxide (DMSO). The microplates were prepared by a liquid handling robotic workstation (Xiril X75), and manually transferred to an automated microplate reader (Chameleon II, Hidex). The reactions were started by automatically injecting NADPH into individual wells and the decrease in absorbance at 340 nm was followed at 37°C for up to 5 min. Samples with added DMSO and no enzyme were included on all plates as controls. Where indicated, bovine serum albumin (BSA) and Triton-X100 were included in the reaction mixtures. For NADPH oxidation at 340 nm a molar extinction coefficient of 6220 M^−1^ cm^−1^ was used. For this assay a *Z*’-factor value of 0.62 was calculated, indicating robustness of the measurement and an acceptable dynamic range of the signal. Primary hits were defined as compounds that inhibited 75 per cent of ThyX activity at the initial screening concentration. The ‘deprotonation’ activity (tritium release assay) of various ThyX proteins was used to confirm the results of the NADPH oxidation assays using published protocols [15,17]. In order to identify inhibitors for crystallization trials, one hundred and sixty 1,4-naphthoquinone (1,4-NQ) derivatives were purchased from Chembridge Corporation along with a control library that consisted of eighty 1,2-naphthoquinone (1,2-NQ) derivatives. The 2-OH-1,4-NQ library (52 molecules) was constructed using molecules purchased from Sigma and Chembridge.

### 3.4. Modality of inhibition

All tests were performed in triplicates in 96-well microtitre plates. A typical assay (200 µl) contained 200 µM NADPH, 5 µM dUMP, 1 mM MgCl_2_, 1 per cent glycerol, 10 µM FAD and 10 μM (total protein concentration) PBCV-1 ThyX. The relatively low dUMP concentration was chosen to favour identification of inhibitors bound to the nucleotide binding pocket of the target enzyme. The concentration of the inhibitors, dissolved in DMSO, ranged between 3.125 and 50 μM. The reactions were started by injection of NADPH. The obtained *V_i_*/*V*_0_ values were plotted for each inhibitor concentration and fitted to sigmoidal dose–response curves using GraphPad Prism 5 software (GraphPad). Morrison *K_i_* values that do not assume that the free concentration of inhibitor equals the total concentration were also determined using GraphPad Prism 5.

ThyX assays (100 μl) under anaerobic conditions contained 77 μM of PBCV-1 ThyX bound FAD in 300 mM NaCl, 1 mM MgCl_2_, 10 per cent glycerol (m/v) and 50 mM HEPES, pH 8.0. Samples were made anaerobic by repeatedly degassing with argon. Traces of oxygen were removed using the glucose oxidase system (60 units of glucose oxidase and catalase in the presence of 10 mM glucose). Where indicated, 100 μM dUMP, 100 μM inhibitor C8-C1 and 400 μM NADPH were added using an air-tight syringe. NADPH oxidation and FAD reduction were followed spectroscopically (optical path length 1 mm) at 340 and 450 nm, respectively.

### 3.5. Testing of anti-microbial activity using genetically modified *Escherichia coli* strains

*Escherichia coli* MG1655 (wild-type, *thyA^+^ tdk^+^*) and FE010 [*ΔthyA::PBCV-1 thyX tdk^+^*] [26] were grown at 37°C either in thymidine-containing LB medium or thymidine-free M9 minimal medium supplemented with 0.1 mM CaCl_2_, 2 mM MgSO_4_ and 0.2 per cent casamino acids (Difco). IPTG-induced overexpression of target protein PBCV-1 ThyX was used to test titration of inhibitor in the bacterial cell. When necessary, thymidine (40 µg ml^−1^) and kanamycin (40 µg ml^−1^) were added to the growth medium. The cells were then harvested and washed two times with M9 medium to remove the remaining thymidine. The cells were diluted to an OD_600_ of approximately 0.1 using M9 medium and 1 ml of cell suspension was transferred to 24-well microplates (Nunc). Compounds were dissolved in DMSO and were added at indicated concentrations. No cells and DMSO-only controls were included for each medium composition. The plates were closed with plastic lids and incubated at 37°C with shaking at 160 r.p.m. After 24 h of incubation, 100 µl of 10-fold serial dilutions were plated onto LB plates or LB plates supplemented with kanamycin (40 µg ml^−1^) when appropriate. Colonies were enumerated after overnight incubation at 37°C. Colony-forming units (CFU) per millilitre for each growth condition were determined from three independent experiments.

### 3.6. Structural analysis of a ThyX–inhibitor complex

Before crystallization, PBCV-1 ThyX (50 mM HEPES pH 8, 300 mM NaCl, 10% glycerol) was incubated with 1 mM FAD as previously described for the crystallization of the PBCV-1 ThyX apo-structure [5]. Co-crystallization with the inhibitor used a 10-fold molar excess of the inhibitors. Figure 4*e* shows crystals that were grown at 19°C from a 1 : 1 μl mixture of a 13 mg ml^−1^ protein solution with a crystallization solution composed of 10 per cent PEG 5000 MME, 12 per cent isopropanol, 12 per cent DMSO, 100 mM MES, pH 6.5*.* For data collection, the crystals were cryo-protected by successive transfers into the crystallization solution supplied with 5 mM of tight-binding C8-C1 inhibitor and increasing ethylene glycol concentrations (final concentration 30% (v/v)) and then flash cooled in liquid nitrogen. The diffraction data were recorded on beam line Proxima-1 (synchrotron SOLEIL, France). The structure was determined by the molecular replacement method using our previously solved crystal structure of PBCV-1 ThyX. Data were processed using the XDS package. The space group was P1 with four ThyX homotetramers per asymmetric unit. The model was refined against a 2.6 Å native dataset using PHENIX and then rebuilt with COOT. The C8-C1 inhibitor could easily be modelled into a residual *F*_o_ – *F_c_* electron density map (contoured at 3*σ*). The Vina AutoDock program was used to predict free energy of binding of the inhibitors at the active site [27]. Where indicated, the residues Gln-75, Gln-188, Arg-90 and Glu-152 (at the interphase of subunits A and C) were made flexible during docking calculations.

## 4. Results

### 4.1. Identification of 2-hydroxyl-1,4-naphthoquinones as selective ThyX inhibitors

In order to identify non-substrate analogue inhibitors of ThyX activity, we screened the National Cancer Institute diversity set (1982 molecules) and a collection of 340 natural compounds (Greenpharma) using the PBCV-1 enzyme (figure 1*c*,*d*, see also [21]). Our initial screen had an approximate success rate of 1 per cent (greater than 75% inhibition at 20 µM (selection criterion 1)) and identified 14 molecules that still inhibited PBCV-1 ThyX activity in the presence of BSA (0.1 mg ml^−1^) and Triton X-100 (0.1%). This observation excluded the possibility that the high success rate of the screen did simply reflect promiscuous inhibition mediated by non-specific protein binding or aggregation. When these 14 molecules were tested using isolated ThyX proteins from *H. pylori*, *C. trachomatis* and *M. tuberculosis,* we identified a single compound, 2-bromo-8-hydroxy-1,4-naphthoquinone (compound 2E04, figure 1*d*) that specifically decreased the activity of all ThyX proteins tested. This compound was originally isolated from the fruit of *Diospyros maritima* and is related to lipid-soluble vitamin K1. No inhibitory effect was observed using human thymidylate synthase (data not shown). This observation was also confirmed by following the loss of tritium from [5-^3^H]dUMP [4,15] during ThyX catalysis, excluding the possibility that results of the colorimetric assay simply reflected inhibition of the non-physiological side reaction with molecular oxygen [26,28]. Additional screenings using a purchased 1,4-NQ library of 160 molecules provided eight ThyX inhibitors including several 2-OH-1,4-NQs (table 1), whereas no ThyX inhibitors were identified using the control library consisting only of 1,2-NQs. Overall, 29 per cent of compounds from a 2-OH-1,4-NQ library acted as ThyX inhibitors.

**Table 1.** Hits issued from screening the 1,4-naphthoquinone libraries.

compound	structure	% inhibition at 20 µM deprotonation	IC_50_ (µM) NADPH oxidation
		PBCV-1 ThyX	
C8-C1 2-hydroxy-3-(4-methoxybenzyl)-1,4-naphthoquinone	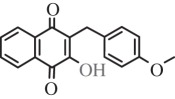	78 ± 4	4.5
E11-C1 2-hydroxy-3-(2-oxo-2-phenylethyl)-1,4-naphthoquinone	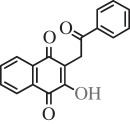	36 ± 5	11.0
H3-C1 2-hydroxy-3-(2-oxo-2-(thiophen-2-yl)ethyl)-1,4-naphthoquinone	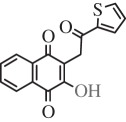	38 ± 3	7
F3-C1 2-hydroxy-3-(2-oxo-2-p-tolylethyl)-1,4-naphthoquinone	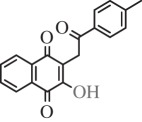	38 ± 7	7
G3-C1 2-hydroxy-3-(2-(4-methoxyphenyl)-2-oxoethyl)-1,4-naphthoquinone	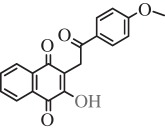	22 ± 7	10
B3-C1 2-(5-hydroxy-1,4-dioxo-1,4-dihydronaphthalen-2-ylamino)benzoic acid	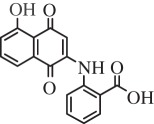	12 ± 6	>10

### 4.2. Determination of the inhibition modality

**Figure 2.**
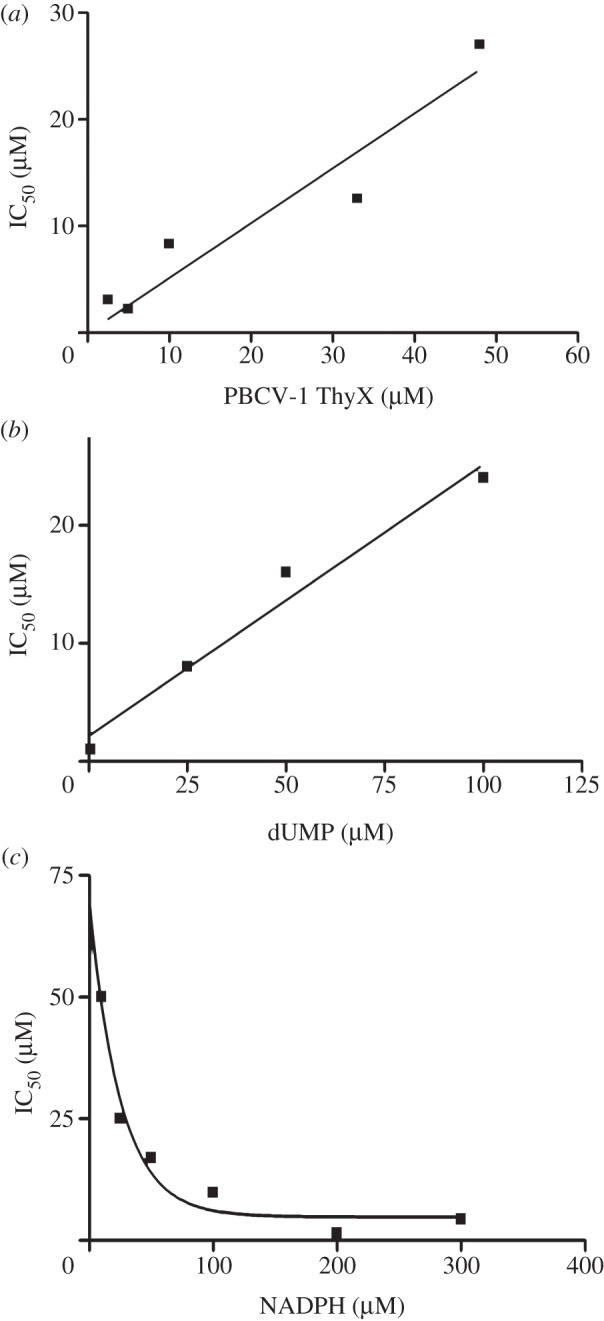
Modality of ThyX inhibition by the molecule C8-C1. (*a*) Variation of the IC_50_ as the function of the PBCV-1 ThyX concentration. The linear relationship is indicative of a tight-binding inhibition mechanism. (*b*) Effect of the dUMP concentration on the IC_50_ value for the molecule C8-C1. (*c*) Effect of the NADPH concentration on the IC_50_ value for the molecule C8-C1 is shown. Data shown are representative of three individual experiments.

As a first step towards the mechanistic understanding of 2-OH-1,4NQs as ThyX inhibitors, we determined the IC_50_ values for a set of identified ThyX inhibitors (table 1). Obtained values were independently confirmed using the ‘deprotonation’ assay at a compound concentration of 20 μM, revealing a robust correlation between measured IC_50_ values and per cent of inhibition determined using NADPH and ‘deprotonation’ assays, respectively. We stress that the total PBCV-1 ThyX concentration used in these assays was 10 μM. Thus, the observation that the IC_50_ values determined from the dose–response curves were similar to the total enzyme concentration used (within a factor of 2–3) suggested a tight-binding inhibition mechanism [29]. In agreement, the IC_50_ value for the molecule C8-C1 varied linearly as a function of the enzyme concentration used in the assay (goodness of fit value *r*^2^ = 0.9125 (figure 2*a*)). When the IC_50_ at a fixed enzyme concentration was plotted as a function of the dUMP concentration (indicated as [S]/km), a linear correlation with a positive slope (*r*^2^ ≈ 0.97) was observed (figure 2*b*), indicating that the molecule C8-C1 acts as a tight-binding competitive inhibitor with respect to dUMP. Using the Morrison equation, we estimate an apparent *K_i_* value of 1.5 μM for the inhibitor C8-C1 that reflects a *K_i_* of 460 nM after correcting for the total enzyme and dUMP concentration used in the assay. This value is in agreement with a *K*_D_ ≈ 400 nM that was determined by C8-C1 induced quenching of FAD fluorescence (J. Briffotaux, S. Laptenok, M. Vos, U. Liebl & H. Myllykallio 2012, unpublished data). An analogous plot for NADPH revealed a characteristic behaviour for tight-binding uncompetitive inhibition (figure 2*c*), with an associated *K_i_* of 4.45 μM.

We also investigated the effect of dUMP and the inhibitor C8-C1 on NADPH oxidation and FAD reduction under anaerobic conditions (table 2). In these experiments, the glucose oxidase system was used to remove traces of molecular oxygen. Our data indicated that in the absence of molecular oxygen and dUMP, NADPH alone was not capable of reducing the FAD cofactor bound to PBCV-1 ThyX. However, when the enzyme was preincubated with dUMP, we observed a rapid FAD reduction that was coupled to NADPH oxidation. Strikingly, we noticed that, differently from the dUMP substrate, the inhibitor C8-C1 did not act as catalytic activator of the viral ThyX protein, further indicating that the inhibitor does not simply act as a substrate analogue.

**Table 2.** Effect of the inhibitor C8-C1 on NADPH oxidation and FAD reduction under anaerobic conditions.

addition	NADPH oxidation (nmol)	FAD reduction (nmol)
NADPH	0	0
dUMP + NADPH	11.3	8.9^a^
C8-C1 + NADPH	0	0^b^

^a^Mean values from two independent experiments are indicated; mean deviation does not exceed 5% of the mean value. Reactions were started by adding dUMP. NADPH oxidation or FAD reduction was not observed using free FAD in the presence or absence of the inhibitor. The enzyme used was PBCV-1 ThyX.

^b^Binding of C8-C1 to the oxidized enzyme was indicated by a red-shift of the FAD spectrum (≈10 nm) after addition of inhibitor.

### 4.3. Anti-microbial activity mimicking a genetic knockout of thymidylate synthase

**Figure 3.**
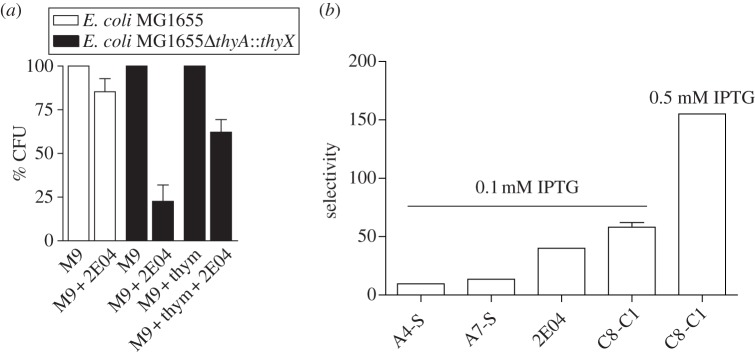
Growth inhibition induced by ThyX inhibitors mimics a genetic knockout of *thyX*. (*a*) Growth inhibition of *E. coli* FE010 (*ΔthyA::thyX tdk^+^*) and the corresponding wild-type strain MG1655 (*thyA*^+^ *tdk^+^*) in thymidine-deprived medium by the lead compound 2E04. The values reported are means of three individual experiments. Hundred per cent refers to cell viability, measured as CFU, in the absence of inhibitor. Note that addition of thymidine reverses growth inhibition, because the *E. coli* strains used can bypass thymidylate synthase activity by salvaging extracellular thymidine. (*b*) Overexpression of *thyX* reduces growth inhibition by the molecules 2EO4 and C8-C1. The selectivity factor refers to the ratio of CFUs observed for the strain FE010 carrying the plasmid overexpressing PBCV-1 *thyX* under an IPTG-inducible promoter with or without induction.

To investigate whether ThyX activity can also be inhibited within the bacterial cell, we used a genetically modified *E. coli* strain in which PBCV-1 *thyX* replaces the chromosomal copy of *thyA* (*E. coli* FE010 *ΔthyA::thyX*)) [30]. Using this strain, we found that the compound 2E04 (2-bromo-8-hydroxy-1,4-NQ, figure 1*d*) at a concentration of 1.5 μg ml^−1^ inhibited, in thymidine-deprived medium, approximately 80 per cent of bacterial growth of *E. coli* FE010, whereas only 10–15% of growth inhibition was observed for the isogenic wild-type strain (figure 3*a*). The only genetic difference between the two strains being the presence of different thymidylate synthase genes, our data indicate direct inhibition of *thyX* in this genetic context. Importantly, this growth inhibition was reversed by the addition of thymidine to the growth medium, which can be explained by the fact that *E. coli* contains thymidine kinase (*tdk*) required for thymidine salvage (figure 3*a*). This cellular phenotype also mimics a genetic knockout of thymidylate synthase. In further support of this notion, we observed that overexpression of ThyX resulted in less-potent growth inhibition, as exemplified by the increased ratio of CFUs (‘selectivity factor’) determined with and without induction for the molecules 2EO4 (1.5 μg ml^−1^) and C8-C1 (50 μg ml^−1^; figure 3*b*). While we cannot fully exclude the possibility of additional cellular targets, these observations indicate inhibition of intracellular ThyX.

### 4.4. Structural basis for inhibition of ThyX activity

**Figure 4.**
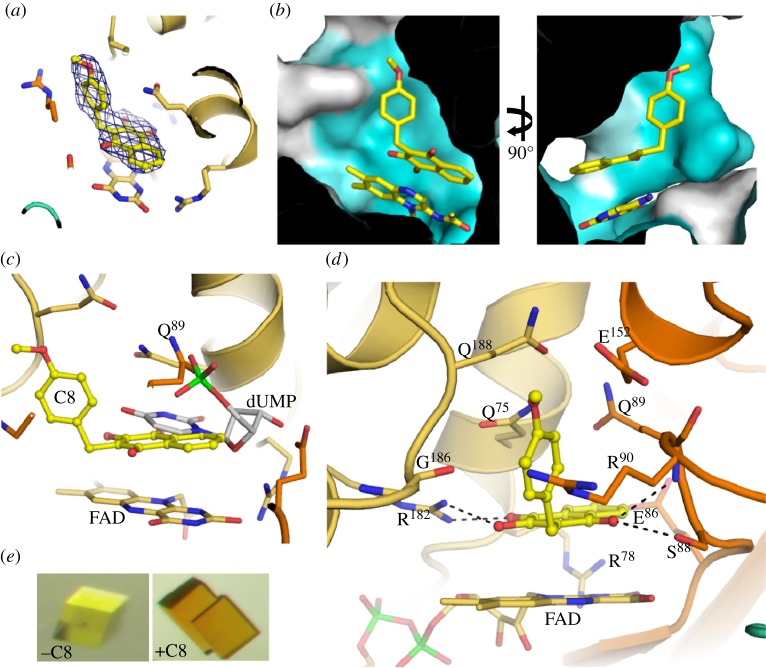
Representation of C8-C1 binding in the PBCV-1 ThyX active site. (*a*) Representation of the C8-C1 inhibitor fitted into the residual *F*_o_ – *F_c_* electron density map contoured at 3*σ*. (*b*) Molecular surface of the sequence conservation among ThyX enzymes. Only the ThyX active site is represented in this view. Colouring is from white (poorly conserved) to blue (highly conserved). The C8-C1 inhibitor and FAD ligand are shown as sticks. (*c*) Comparison of the dUMP (grey sticks) and C8-C1 (yellow ball and sticks) binding modes in the ThyX active site. dUMP has been modelled by superimposing the crystal structure of *T. maritima* ThyX bound to dUMP onto the current structure (r.m.s.d. = 1.7 Å over 180 Cα) [6]. FAD is shown in sticks. (*d*) The C8-C1 inhibitor bound at the ThyX active site is shown as ball and sticks. ThyX monomers A and B are coloured beige and orange, respectively. Black dashed lines depict hydrogen bonds. The presence of four copies of the ThyX homotetramer in the crystal asymmetric unit allowed us to establish that in 16 distinct active sites, the inhibitor binds in a very similar way. (*e*) Crystals of PBCV-1 ThyX obtained in the absence (yellow) or presence (dark orange) of compound C8-C1. The change in colour indicates binding of C8-C1 to the oxidized (yellow) form of the enzyme.

In order to obtain further insight into the mechanism of ThyX inhibition, we solved the crystal structure of C8-C1 bound to PBCV-1 ThyX at a resolution of 2.6 Å. We observed residual *F*_o_ – *F_c_* electron density, which perfectly accommodated the C8-C1 compound (figure 4*a* and table 3). C8-C1 binds within the catalytic pocket between two ThyX monomers (hereafter called monomer A and B). The C8-C1 binding site is conserved (figure 4*b*) and partially overlaps with the dUMP binding pocket observed in *T. maritima* and *M. tuberculosis* ThyX [6,8]. These observations provide a plausible structural explanation why C8-C1 inhibits all ThyX enzymes tested. The 1,4-NQ moiety binds in the same cavity as the uracil ring from dUMP and is sandwiched between the FAD isoalloxazine ring on one side and side chains from Gln-75 (monomer A) and Gln-89 (monomer B) on the other side (figure 4*d*). The binding of the 1,4-NQ moiety is further stabilized by four hydrogen bonds: the carbonyl group at position 4 interacts with the hydroxyl group from Ser-88 and the main chain N from Gln-89 in monomer B. On the opposite side of the ring, the Arg-182 side chain from monomer A forms hydrogen bonds with the 1,4-NQ carbonyl and hydroxyl groups at positions 1 and 2, respectively. The aliphatic parts of the Gln-75, Glu-152 and Arg-90 side chains form the wall of a hydrophobic pocket that accommodates the 4-methoxybenzyl group of C8-C1. This pocket is distinct from the one accommodating the dUMP phosphoribose moiety; hence, the binding mode of the inhibitor is distinct from dUMP binding. Comparison with the structure of the FAD-bound form of PBCV-1 ThyX shows that upon inhibitor binding, a long loop located in the vicinity of the substrate binding site (residues 89–104) becomes ordered and participates in C8-C1 binding. In addition, the Gln-89 side chain occupies the dUMP phosphate binding pocket (figure 4*c*). Our structural data also explain the importance of the hydroxyl moiety at position 2 of 1,4-NQ for inhibitor binding. This group is hydrogen-bonded to the side chain of the strictly conserved Arg-182 from monomer A.

**Table 3.** X-ray data collection and refinement statistics. Values in parentheses are for highest resolution shell.

	native
resolution (Å)	35–2.6 (2.75–2.6)
space group	P1
cell parameters	*a* = 70 Å; *b* = 120.6 Å; *c* = 128 Å; *α* = 111.6°; *β* = 91.2°; *γ* = 90.0°
total number of reflections	252 855
total number of unique reflections	111 996
*R*_sym_ (%)^a^	6.9 (52.4)
completeness (%)	92.6 (93.4)
*I*/*σ*(*I*)	10.7 (1.8)
redundancy	2.25
*refinement*	
resolution (Å)	35–2.6
*R*/*R*_free_ (%)^b^	21.1/26.3
r.m.s.d. bonds (Å)	0.010
r.m.s.d. angles (°)	1.19
*Ramachandran plot*	
most favoured (%)	93.1
allowed (%)	6.9
disallowed (%)	0
PDB code	4FZB

^a^*R*_sym_ = *Σ_h_Σ_i_*|*I_hi_* − 〈*I_h_*〉|/*Σ_h_Σ*_i_*I_hi_* where *I_hi_* is the *i*th observation of the reflection *h*, while 〈*I_h_*〉 is the mean intensity of reflection *h*.

^b^*R*_factor_ = *Σ*||*F_o_*| − |*F_c_*||/|*F_o_*|. *R*_free_ was calculated with a small fraction (5%) of randomly selected reflections.

**Figure 5.**
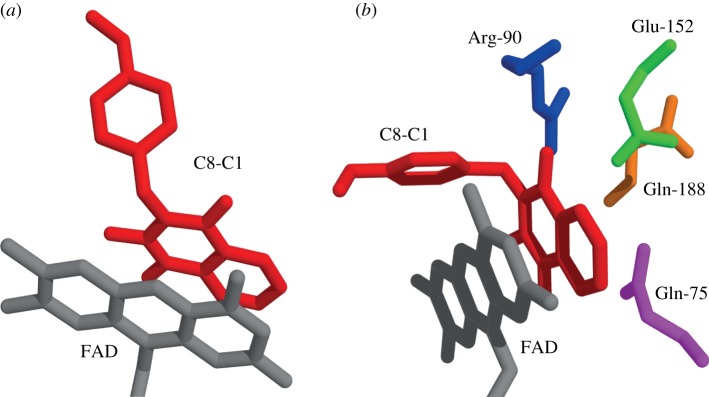
Predicted docking poses of ThyX inhibitors in the active site of ThyX. (*a*) The rigid docking pose of the molecule C8-C1 stabilized by stacking interactions with the FAD and the residues indicated below. (*b*) The docking pose obtained when amino acid residues Gln-75, Gln-188, Arg-90 and Glu-152, at the interface of subunits A and C, were made flexible during docking calculations. Note that conformational flexibility of these residues may play a key role in directing the binding of 2-OH-1,4-NQs at the active site. The picture was prepared using OpenAstexViewer 3.0.

Predicted docking poses of ThyX inhibitors in the active site of ThyX were obtained using the Vina AutoDock program and are indicated in figure 5 using rigid (figure 5*a*) and flexible (figure 5*b*) docking. Note that conformational flexibility of the amino acid residues Gln-75, Gln-188, Arg-90 and Glu-152, at the interphase of subunits A and C, may play a key role in directing the binding of 2-OH-1,4-NQs at the active site. Note also that these calculations suggested a free binding energy of −10.5 ± 2.75 kcal mol^−1^ for the molecule C8-C1, in agreement with the values of −8.61 and −8.69 kcal mol^−1^ calculated using the experimentally determined *K_i_* and *K_d_* values, respectively.

## 5. Discussion

We identified 2-hydroxy-1,4-naphthoquinones as potent ThyX inhibitors and demonstrated that they bind to the evolutionary conserved and flexible active site of ThyX proteins in a unique configuration. Our structural data indicate that the observed binding mode of the C8-C1 inhibitor is reminiscent of the stacking of a substrate quinone moiety onto the FAD isoalloxazine group that was previously observed for human and rat NADPH-quinone reductases [31,32]. However, the inhibitory mechanism of our inhibitors is clearly distinct from what has been previously described for a clinically used anti-malarial compound atovaquone, 2-[trans-4-(4-chlorophenyl)cyclohexyl]-3-hydroxy-1,4-NQ, that binds *in vitro* to ubiquinone binding sites of the cytochrome *bc*_1_ complex [33] and the class II dihydroorotate dehydrogenase [34]. Steady-state kinetic measurements and docking studies indicated high affinity of our inhibitors towards ThyX proteins. We observe that this compound can bind to the oxidized enzyme with high affinity, thus preventing binding of dUMP and the reduction of the FAD cofactor, both of which are necessary for enzyme function. The tight-binding inhibitory mechanism we have demonstrated is analogous to earlier studies that have demonstrated that for instance methotrexate binds to the free dihydrofolate reductase and the enzyme–NADPH complex, but shows higher affinity for the latter [35]. Our steady-state kinetic analyses also indicate that dUMP and NADPH/folate binding sites of ThyX proteins do not considerably overlap, in agreement with recent structural studies [18]. Altogether, our biochemical and structural observations suggest a molecular regulatory mechanism that allows ThyX protein to oxidize NADPH only when the nucleotide substrate is bound to the active site of the protein, and thus diminishing unwanted side reactions with molecular oxygen.

Notably, our data indicate intracellular targeting of PBCV-1 ThyX protein, which is in agreement with the relatively low *K_i_* and *K_d_* values of the molecule C8-C1. Our experiments with genetically modified *E. coli* strains also exclude the possibility that the anti-microbial activity of our compounds simply results from non-specific redox and/or arylation reactions. It is also of note that 2-substituted 1,4-naphthoquinones do not participate in lipid peroxidation and formation of mutagenic DNA base adducts such as 8-hydroxy-2′-deoxyguanosine [36]. The observation that 2-OH-1,4-NQs are well tolerated [37] and, in the case of atovaquone, have passed clinical trials is likely related to their incapability to readily form a semiquinone radical [33].

In conclusion, our inhibitory screen revealed the first tight-binding ThyX inhibitors that do not simply act as substrate analogues. Notably, tight-binding inhibitors may provide benefits for the possible clinical use of ThyX inhibitors owing to the relatively long residence time of the compounds on the target enzyme. Our observations reported here and the wide interest in ThyX as an anti-microbial target encourage further identification and optimization of ThyX inhibitors towards possible biomedical use.

## 6. Acknowledgements

We thank S. P. Laptenok and M. Vos for helpful discussions. This work was supported by ANR projects THYMET and AMTHYX. H.M. also acknowledges financial support from the foundation Bettencourt-Schueller.

## Supplementary Material

Basta et al supplemental material
