# Supplementary material for: Mechanistic and structural basis for inhibition of thymidylate synthase ThyX
Source: Open Biol. 2012 Oct;2(10):120120. doi: 10.1098/rsob.120120 (PMC3498832; doi:10.1098/rsob.120120)
Supplement: Basta et al supplemental material [file rsob120120-s1.docx]

**Table S1**: Human pathogenic bacterial species carrying ThyX (non-exhaustive list) and their epidemiological impact

| Bacterial species | Associated diseases | Incidence/comments* | Tdk^$^ | Lifestyle |
| --- | --- | --- | --- | --- |
| *Bacillus anthracis* | anthrax | potential biological warfare agent | + | aerobic |
| ***Borrelia burgdorferi*** | **Lyme disease** | **about 70000 reported cases/year in Europe** | **+** | **microaerophilic** |
| *Campylobacter jejuni* | diarrhea | estimated 400 million cases worldwide | - | microaerophilic |
| ***Chlamydia trachomatis*** | **trachoma** | **estimated 500 million people at high risk of infection, over 140 million persons are infected worldwide** | **-** | **microaerophilic** |
| *Chlamydia pneumoniae* | pneumonia | the overall incidence is unknown, an estimated 2-5 million cases of pneumonia and 500,000 pneumonia-related hospitalizations in the U.S.A. each year | - | microaerophilic |
| *Clostridium botulinum* | botulism | rare incidence, mortality rate is high, the disease can be fatal in 5 to 10% of cases | + | anaerobic |
| *Clostridium difficile* | diarrhea, colitis | estimated global death toll from diarrhoeal diseases is about two million deaths/year worldwide | - | anaerobic |
| *Clostridium tetani* | tetanus | 290,000 estimated deaths (2000-2003) worldwide | + | anaerobic |
| *Corynebacterium diphteriae* | diphtheria | 4190 reported cases worldwide in 2007 | - | aerobic |
| ***Helicobacter pylori*** | **stomach ulcer, gastric cancer** | **an estimated prevalence of about half the world’s population** | **-** | **microaerophilic** |
| *Leptospira interrogans* | leptospirosis | estimated 0.1 – 1/100 000 people living in temperate climates are affected each year, and 10 or more per 100 000 people living in tropical climates | - | aerobic |
| *Mycobacterium leprae* | leprosy | 254,525 of new cases detected during 2007 worldwide | - | aerobic |
| ***Mycobacterium tuberculosis*** | **tuberculosis** | **one-third of the world's population is currently infected, estimated 1.6 million deaths in 2005.** | **-** | **aerobic** |
| *Mycobacterium bovis* | bovine tuberculosis | the share of bovine tuberculosis in global epidemic is not known, recent work indicates that this may be a substantial fraction | - | aerobic |
| *Rickettsia prowazeki* | typhus | potential for explosive endemics in man | - | microaerophilic |
| *Rickettsia rickettsii* | spotted fever | about 250-1250 new cases/year reported in U.S.A. | - | microaerophilic |
| *Treponema pallidum* | syphilis | about 11000 new cases each year in U.S.A. | - | microaerophilic |

*Data were obtained from world health organization [www.who.org](http://www.who.org) and Centers for Disease Control and Prevention [www.cdc.org](http://www.cdc.org)

^$^occurrence of thymidine kinase required for thymidine salvage in bacterial genomes as indicated in Clusters of Orthologous Groups (COG) database: <http://www.ncbi.nlm.nih.gov/COG/grace/wiew.cgi?COG1435>
